# Similar Local and Landscape Processes Affect Both a Common and a Rare Newt Species

**DOI:** 10.1371/journal.pone.0062727

**Published:** 2013-05-03

**Authors:** Mathieu Denoël, Amélie Perez, Yves Cornet, Gentile Francesco Ficetola

**Affiliations:** 1 Laboratory of Fish and Amphibian Ethology, Behavioural Biology Unit, Department of Biology, Ecology and Evolution, University of Liège, Liège, Belgium; 2 Geomatics Unit, University of Liège, Liège, Belgium; 3 Department of Environmental Sciences, University of Milano-Bicocca, Milano, Italy; Universität Zurich, Switzerland

## Abstract

Although rare species are often the focus of conservation measures, more common species may experience similar decline and suffer from the same threatening processes. We tested this hypothesis by examining, through an information-theoretic approach, the importance of ecological processes at multiple scales in the great crested newt *Triturus cristatus*, regionally endangered and protected in Europe, and the more common smooth newt, *Lissotriton vulgaris*. Both species were similarly affected by the same processes, i.e. suitability of aquatic and terrestrial components of their habitat at different scales, connectivity among breeding sites, and the presence of introduced fish. *T. cristatus* depended more on water depth and aquatic vegetation than *L. vulgaris*. The results show that environmental pressures threaten both common and rare species, and therefore the more widespread species should not be neglected in conservation programs. Because environmental trends are leading to a deterioration of aquatic and terrestrial habitat features required by newt populations, populations of the common species may follow the fate of the rarest species. This could have substantial conservation implications because of the numerical importance of common species in ecosystems and because commonness could be a transient state moving towards rarity. On the other hand, in agreement with the umbrella species concept, targeting conservation efforts on the most demanding species would also protect part of the populations of the most common species.

## Introduction

An important question in conservation biology is whether sympatric rare and common species can be similarly affected by habitat change [1]. Because they are more abundant or have a broader range of distribution, common species are often believed to be not threatened. Until recently, they have also attracted less attention from ecologists, a consequence of their less preoccupying conservation status [2]. In this perspective, they were thought to indirectly take advantage of the protection of rare species through the umbrella species concept, even though this would require a sufficient distribution overlap and similar minimum requirements as the rarest species [3]. However, because of their numerical importance in ecosystems, and consequently their large contribution to the global biomass, it is now recognized that status changes of common species may have important consequences [4]. Furthermore, over geological times and space, commonness is only a transient situation [2].

The conservation of rare species can allow the conservation of common species if they are similarly affected by threatening processes [5]. In such situations, the rarest or most threatened species are expected to be more severely affected by environmental processes than the more common ones. Identifying the threatening processes is challenging [6], but the formulation of *a priori* hypotheses on ongoing processes, followed by the application of information-theoretic statistical models, explicitly testing these hypotheses, can greatly help the identification of threatening processes based on distribution patterns [7].

**Figure 1:**
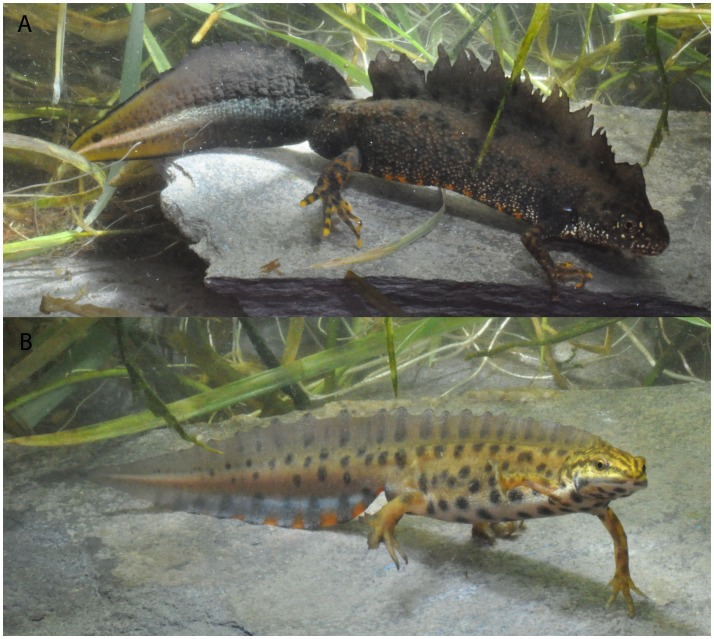
The crested newt (*Triturus cristatus*) (A) and the smooth newt (*Lissotriton vulgaris*) (B). Both pictures show males from a pond in Pays de Herve (Belgium) and are representative of a rare and emblematic (A) and a more common and less protected (B) species.

Amphibians are a valuable group in which to examine these questions as they are one of the most threatened classes of organisms worldwide, but also because much attention has been paid to the rarest species [8], [9]. Common amphibian species also face population declines, such as the common toad (*Bufo bufo*) in Europe and the northern leopard frog (*Lithobates pipiens*) in many states of the USA [10], [11]. In newts, several conservation programs (e.g. Life, Natura 2000) have focused on the great crested newt *Triturus cristatus*, an emblematic species protected under the Habitat Directive Annex 2 [12]–[17] (Figure 1A). In contrast, the smooth newt *Lissotriton vulgaris* (Figure 1B) is much less protected, in part because of its assumed commonness. However, reports of regional decline suggest that it could also be affected by environmental pressures [17]–[19].

Previous research on crested and smooth newts has improved our knowledge on the ecological requirements of these species, but also raised new questions (see e.g. [1], [15], [20], [21]). In particular, most research undertaken on both crested and smooth newts living in sympatry occurred in areas where they remained widely distributed and where both species were equally frequent in ponds (see Table 1 for details [1], [21]–[25]). The situation remains to be clarified in more modified agricultural landscapes where the crested newt is much rarer than the smooth newt. This pattern is typical of Western Europe, where modernization of agricultural practices and urbanization of natural lands has resulted in a decline of pond-breeding amphibians [26], [27]. Therefore, this situation may be representative of very large areas of Europe in the near future. Two previous studies fit this pattern, but have suggested that new surveys targeting sympatric crested and smooth newts, including a larger set of variables, are needed to identify processes acting at various scales [22], [23]. Specifically, a number of previous studies considered landscapes within a radius of 400–500 m from ponds, whereas finer-grain studies suggest that more detailed, shorter-range analyses may also be valuable [28]. Among the wide range of pollutants that are toxic to amphibians, laboratory studies have evidenced the risk of water pollution by nitrogenous compounds as found in fertilizers and urban water discharges [29], but until now no field studies have assessed their detrimental effect on newt distribution. This could be particularly relevant in periurban and agricultural areas dominated by cattle grazing. Past landscapes (i.e. historical land use) have also not yet been examined in newts, although they could also affect these species today [30].

**Table 1:** Summary of landscape ecology studies on sympatric *Triturus cristatus* and *Lissotriton vulgaris*: sampling, geography, and important variables.

Study	Country	*N*	Stat.	% Species occurrence and important variables
[23]	U.K.	203	M	*T.c.* (8%): scrub (+), tertiary deposits (+), greensands (+), fish (−)
				*L.v.* (32%): tertiary deposits (+), scrub (+), gardens (+), chalk (+)
[24]	U.K.	20	U	*T.c.* (55%): pond area (+)
				*L.v.* (43%)
[22]	Belgium	258	M	*T.c.* (5%): depth (+)
				*L.v.* (27%): urban cover (−), distance to forest (+), depth (+), pond area (+), fish (−)
[21]	Romania	54	M	*T.c.* (52%): forest distance (−)
				*L.v.* (43%): high vehicular traffic (-)
[1]	Denmark	210	U	*T.c.* (47%): uncultivated lands (+), sand (+), clear water (+), management (+)
				*L.v.* (65%): uncultivated lands (+), sand (+), clear water (+), management (+), fish (−)
			M	*T.c.* (47%): open lands+forest (+), distance to pond (−), invertebrate diversity (+)
				*L.v.* (65%): sand (+), distance to pond (−), invertebrate diversity (+)
[25]	Norway	207	M	*T.c.* (13%): forest distance (−), pH (−), Chloride-Calcium (+), aquatic vegetation (+), fish (−), occurrence *L.v.* (+)
				*L.v.* (15%): forest distance (−), pH (+), Chloride (+), occurrence *T.c.* (+)
[39]	Switzerland	87	M	*T.c.:* pond permanence (+), fish density (−)*, forest cover (−)
				*L.v.*: predation risk, abundance of other newts (+), forest cover (−), urban cover (−)
			M	*T.c.*: abundance of other newts (+), fish density (−)*
				*L.v.*: aquatic vegetation (+), abundance of other newts (+), forest cover (−)

*N* = number of sampled ponds, Stat.: statistics (U: univariate, M: multivariate), *T.c.*: *Triturus cristatus* (crested newt), *L.v. Lissotriton vulgaris* (smooth newt).

* In this study, *T. cristatus* never co-occurred with fishes.

The objective of this study was to identify the major processes (Table 2) that could threaten these two newt species at different scales in an agricultural landscape that has been affected by habitat change over the last few decades [31]. We hypothesize that both species rely similarly on environmental conditions, thus validating the umbrella species concept, that they are simultaneously affected by multiple processes, and that the rarest species could be declining more rapidly because of greater sensitivity to habitat degradation.

**Table 2:** Variables used for ecological modelling of spatial variation in newt abundance.

	Processes	Environmental variables	PCA results		
			local_1	local_2	local_3
**1**	Suitability of pond (local) features	NO_2_ concentration^a^	**0.671**	−0.043	−0.165
		NH_4_ concentration^a^	**0.782**	−0.029	0.245
		PO_4_ concentrataion^a^	**0.737**	−0.049	−0.282
		O_2_ concentration^a^	−**0.668**	0.090	−**0.395**
		Pond area^a^	0.138	**0.881**	0.059
		Max. depth	−0.323	**0.753**	−0.138
		% aquatic vegetation^b^	−0.049	−0.036	**0.904**
**2**	Fish presence	Fish presence	–		
**3a**	Connectivity (100 m)	*N* wetlands within 100 m^c^	–		
**3b**	Connectivity (500 m)	*N* wetlands within 500 m^c^	–		
			land_100m_1	land_100m_2	
**4a**	Present landscape composition (100 m)	% garden^b^	**0.926**	0.136	
		% cultivated land^b^	−0.046	**0.786**	
		% woodland^b^	0.030	−**0.706**	
		*N* buildings^c^	**0.891**	−0.260	
			land_500m_1	land_500m_2	
**4b**	Present landscape composition (500 m)	% garden^b^	**0.954**	0.001	
		% cultivated land^b^	−**0.457**	**0.722**	
		% woodland^b^	−0.197	−**0.889**	
		*N* buildings^c^	**0.958**	−0.050	
			past_100m_1	past_100m_2	
**5a**	Past landscape composition (100 m)	% garden^b^	**0.889**	0.164	
		% cultivated land^b^	0.125	**0.750**	
		% woodland^b^	0.191	−**0.777**	
		*N* buildings^c^	**0.886**	−0.239	
			past_500m_1	past_500m_2	
**5b**	Past landscape composition (500 m)	% garden^b^	**0.911**	0.175	
		% cultivated land^b^	−0.128	**0.718**	
		% woodland^b^	−0.136	−**0.852**	
		*N* buildings^c^	**0.866**	−0.184	

Ecological processes that can threaten newt species, variables and results of principal component analyses (PCAs) summarizing them in a lower number of uncorrelated components.

a log-transformed,

b square-root arcsine-transformed,

c square-root transformed.

In bold, significant correlations with PCA components after Bonferroni’s correction (*α*′ = 0.0009).

## Materials and Methods

### Ethics Statements

The capture permit was authorized by the Ministère de la Région Wallonne (Division de la Nature) and issued on 19th February 2008 on the basis of ethics approval of the field study on newt ecology by Conseil Supérieur Wallon de la Conservation de la Nature.

### Study Area and Sampling Procedures

**Figure 2:**
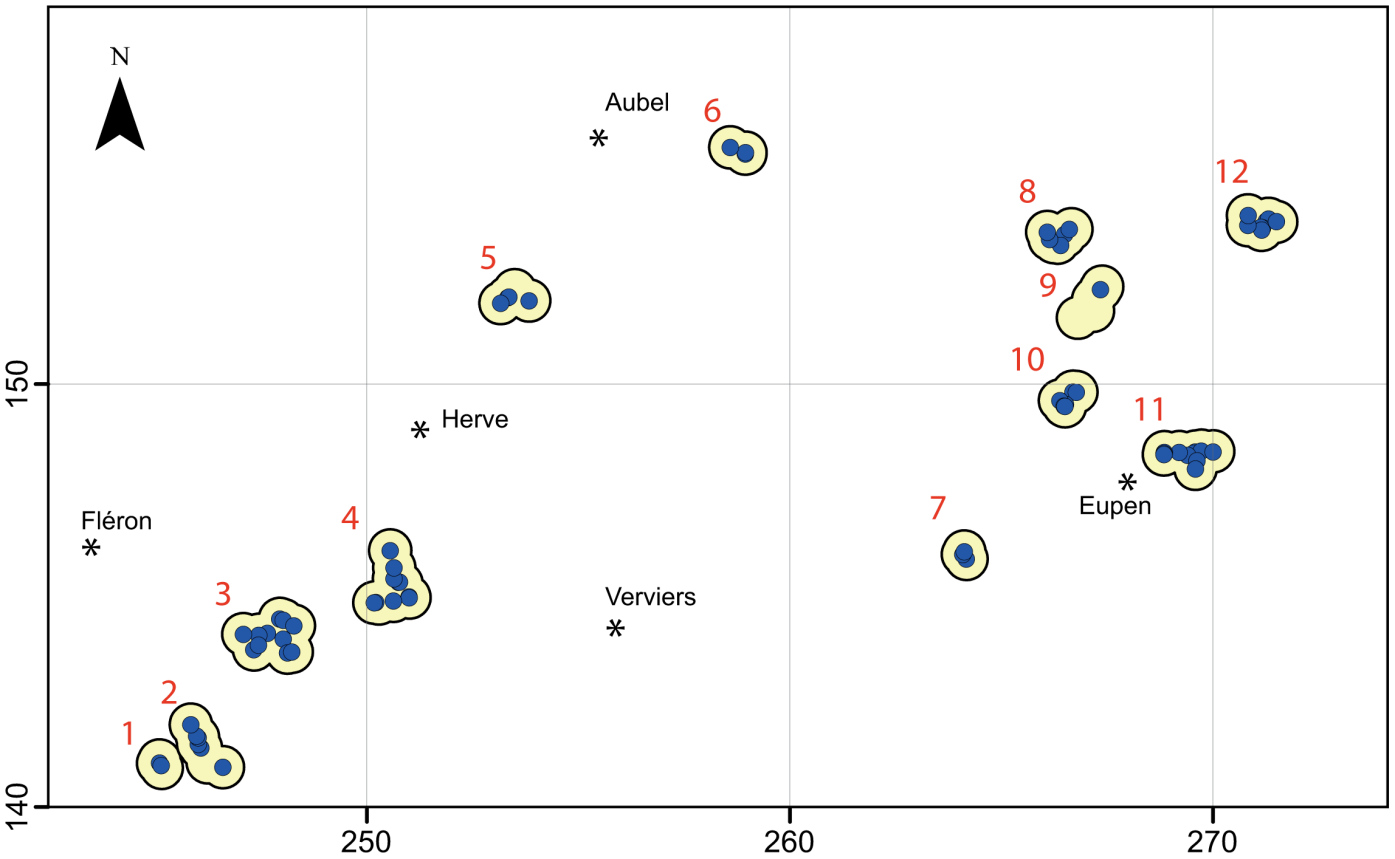
Location of studied ponds in Pays de Herve (Belgium). Blue circles: ponds, yellow patches: studied localities (based on historical presence of *Triturus cristatus*): 1, En Géliveau; 2, Hansez; 3, Haute Rafhay; 4, Stoki; 5, Margarins; 6, Vogelsang; 7, Blanc Baudet; 8, Gut Benesse Hof; 9, Hof Krompelberg; 10, Gemerhet; 11, Corney; 12, Harbenden. Geographic coordinates: Belgian Lambert Grid (expressed in km).

This study was conducted in Pays de Herve, an agricultural area of eastern Belgium at the border of Germany and the Netherlands. The sampling areas were chosen from the known distribution of the crested newt [22]. We surveyed 74 ponds during the newt reproductive season (March–June 2008) (Figure 2). The surveys took place in the 12 ponds historically inhabited by this species (1990–2004), in all ponds within a 500-m radius around these ponds, and also within a 500-m radius around these new sets of surrounding ponds (Figure 2). Surrounding ponds were located using recent topographic maps (Institut Geographic National [IGN], 1∶20,000, published in 1999–2000), colour orthoimages (IGN: Direction Générale de l’Agriculture, 0.5-m resolution, 2006), and by field observations.

To obtain an index of newt abundance, each pond was sampled by dip-netting (40×35 cm dip-net with a 1.8 m pole) [32]. All ponds were visually screened for newts at the time of dip-netting but no more species were found in this way. The small size of ponds allowed covering the entire surface of the ponds several times. Deep zones were additionally sampled with large nets (5 to 10 m^2^ seines). This sampling design was particularly adapted to reduce escape possibilities during netting. Censuses ended after several unsuccessful nettings in various areas of the ponds, including open and vegetated parts. This method has the advantage of sampling all micro-habitats and thus gives comparable values across ponds since all ponds were surveyed similarly. Previous studies showed that the detection probability with this type of removal estimate is very high [33] and that using dip-nets is an adequate method for sampling European newts [34]. Because previous studies highlighted that amphibians, including newts, can leave water temporarily during the breeding season [35], [36], our method did not aim to determine the total adult population, but rather to approach the size of the aquatic adult population as closely as possible at any given time. By sampling all habitats, including pond vegetation and banks, hidden newts can also be captured. Previous studies have shown that blind dip netting such as done in the present study does not give lower abundance estimates during daytime than during night-time [37]. Although it is possible that one species was missed in some ponds, the absence of a record indicates that the species is very rare in that pond or suggests that this would at best be a “sink” or transient habitat [38], [39]. Previous studies have shown that newt abundance is correlated with habitat quality [32]. We therefore believe that our approach is sufficiently robust to evaluate the association between species and environmental variables. We took into consideration only adults because we sought to determine the index of abundance similarly across ponds. There were no ponds in which we found larvae and no adults (qualitative checks were carried out at other times during the study period). All amphibians were handled with wet gloves during sampling. All material was washed and disinfected after every visit to a pond.

### Pond and Landscape Traits

We measured several environmental variables representing five major processes that can determine newt distribution (Table 2). Five variables describing pond features were measured *in situ* during the newt census (Table 2). The maximum water depth and macrophyte cover were assessed in the field, whereas the pond surface area was assessed either in the field or obtained through aerial photo interpretation. The presence of introduced fish (both native and exotic to Belgian fauna, but all outside their natural habitat) was determined by dip-netting and seining, visual observations, and interviewing local owners. These fish are often locally invasive. Oxygen was measured with an oximeter (Hach Lange Multi HQ40d). To evaluate water pollution, a specific visit of each pond was made to gather water samples, which were preserved at 4°C and directly transported to the laboratory for chemical analyses. Sampling took place within 2 days in June. The concentration of three nutrients, mostly caused by water pollution (orthophosphates, nitrites and ammonium) was evaluated through colorimetric analysis using, respectively, blue Molybedne, de Griess and Berthelot reagents.

The number of ponds within 100 and 500 m of the focal pond was recorded as a measure of the pond’s present-day connectivity (Table 2). These values were chosen because radio-tracking studies showed that 50% of movements occurred within 100 m, whereas 500 m is usually considered to cover most movements [20], [40]. Connectivity was available for the present-day period only, because not all ponds were recognizable on historical maps. To evaluate landscape composition, shape files were drawn from recent orthoimages (IGN – DGA, 2006) and historic aerial images (IGN, 1947–1954) in ArcGis 9.3 (Esri, Redlands, CA, USA) to represent land cover limits (i.e. forest, croplands, gardens, buildings and ponds). First-order polynomial functions were adjusted using ground control points selected on the scanned historical aerial images and on the 2006 reference orthoimages. Topographical maps and field visits helped create the landscape layers.

### Statistical Analyses

Three out of five “processes” were represented by multiple environmental variables (Table 2), which were strongly correlated to each other. Including correlated variables may bias the regression results; preliminary models including the original variables showed high values of the variance inflation factor, indicating that multicollinearity affected these models. We therefore used principal component analysis (PCA) to summarize variables in a lower number of uncorrelated components. PCA was performed using the correlation matrix and variable scaling. To keep the roles of these five processes distinct, we performed separate PCAs for the variable set representing each one (Table 2). Extracted components were rotated (Varimax rotation) to improve interpretation. The PCA was run over pond features, present-day and historical pond landscapes at both the 100 and 500 m radius. Extracted components explained 67%, 71%, 85%, 72%, and 74% of the total variance. The correlation between the original variables and the extracted components is shown in Table 2.

We used an information-theoretic approach, based on Akaike’s information criterion (AIC), to identify the processes and the spatial scales most likely to affect the abundance index of the two newt species [7], [41]. We analysed relationships between newt abundance and environmental features using generalized linear models (GLMs), assuming a quasi-Poisson error distribution to take into account overdispersion. First, we built GLMs considering all possible combinations of the variables (either environmental variables or PCA components) representing the five processes (Table 2). For each model, we calculated the quasi-AIC corrected for small sample size (Q-AICc) [42]. A model was not considered as a “candidate model” if a simpler, nested model had a lower Q-AICc [43]. Furthermore, for each process, we considered only one spatial scale at a time, meaning if we included connectivity at the 100 m scale, we did not include connectivity at the 500 m scale and vice-versa. For each candidate model *i,* we then calculated the Q-AICc weight *w*_i_, which is the probability that a given model is the best one, given the set of candidate models considered [44]. We also reported significance values of variables included in the best models, to facilitate the interpretation of the models and of the role played by predictors [45]. Using Q-AIC instead of Q-AICc would not change the results (for both species the best models would remain the same). Errors were not spatially autocorrelated (for all best models, Moran’s *I* <0.05, *P*>0.2). None of the candidate models showed multicollinearity (for all models and all variables, variance inflation factor <5). Conditional partial regression plots were built using the visreg package [46]. Finally, we used an unequal variance *t*-test to compare the features of ponds inhabited by the rarest species or only by the common species [47].

## Results

*Triturus cristatus* was found in 16% of the ponds (*n* = 12 out of 74) within the known area of presence of the species. Six out of the 12 ponds inhabited by *T. cristatus* were not the same as the ones detected in the 2004 study. The average number of adults detected in inhabited ponds (± SE) was 7±3; in the most populated pond, we detected 32 adults. *L. vulgaris* was found in 45% of the ponds (*n* = 33 out of 74). In ponds inhabited by *L. vulgaris*, the average number of adults detected was 24±5 and the maximum number detected in a given pond was 123.

*T. cristatus* was not found in 6 of the 12 historical sites and *L. vulgaris* in 2 out of the 10 historical sites. One of the ponds had disappeared because of the construction of a railway. The others were still present. *L. vulgaris* was observed in one pond where it was not detected during the past census. The new survey allowed the addition of six new populations of *T. cristatus* in sites that were not surveyed in the previous study.

**Figure 3:**
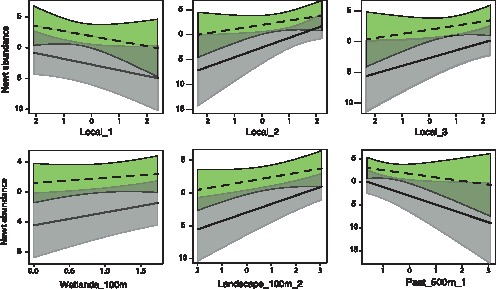
Effect of local and landscape variables on spatial variation in newt abundance in ponds. Panels represent conditional partial regression plots, based on the best selected model for both *Triturus cristatus* (grey bands and full lines) and *Lissotriton vulgaris* (green bands and interrupted lines). The “number of wetlands” were square-root transformed values; the other variables are components extracted by PCAs: see methods for more details. Shaded areas represent 95% confidence bands.

The model that most likely explained the distribution of *T. cristatus* (i.e. the model with rank 1 in Table 3) suggests that this species is influenced by present landscape features (scale, 100 m), past landscape (scale, 500 m), fish presence, local features and connectivity (scale, 100 m) (Tables 3 and 4a). Three further models showed weight greater than 0.1. All the models with weight greater than 0.1 included local features, connectivity and present and past landscape composition (Table 3). Fish presence was included in three out of the four models with weight greater than 0.1. Examination of individual variables included in the best AIC model showed that *T. cristatus* was associated with large and deep ponds with abundant aquatic vegetation but without fish, surrounded by a high number of wetlands within 100 m, in landscapes that are currently open and that had low urbanization in the past (Figure 3, Table 4a).

**Table 3:** Candidate models explaining spatial variation in abundance of *Triturus cristatus* on the basis of ecological variables.

Rank	Model structure	K	Q-AICc	weight
1	Present landscape (100 m), past landscape (500 m), fish presence, suitability of pond features, connectivity within 100 m	10	46.01	0.410
2	Present landscape (100 m), past landscape (100 m), fish presence, suitability of pond features, connectivity within 100 m	10	47.74	0.173
3	Present landscape (100 m), past landscape (500 m), suitability of pond features, connectivity within 100 m	9	47.87	0.162
4	Present landscape (100 m), past landscape (100 m), suitability of pond features, connectivity within 100 m	9	48.36	0.127
5	Present landscape (100 m), fish presence, suitability of pond features, connectivity within 100 m	8	50.65	0.040
6	Past landscape (100 m), suitability of pond features, connectivity within 100 m	7	50.85	0.036
7	Present landscape (100 m), past landscape (500 m), fish presence, suitability of pond features	9	51.08	0.033
8	Present landscape (100 m), suitability of pond features, connectivity within 100 m	7	53.32	0.011

Only models with weight >0.01 are shown here. K = number of estimated parameters.

**Table 4:** Regression coefficients for the processes involved in the spatial variation in newt abundance.

Variables		*β*	95% CI		df	*F*	*P*	Characteristics
a. *Triturus cristatus*								
	Past_500_1	−1.92	−3.80	−0.53	1	8.3	**0.006**	Low urbanization in the past
	Past_500_2	0.83	−0.20	2.37	1	2.4	0.127	
	Landscape_100_1	0.57	−0.27	1.76	1	1.6	0.218	
	Landscape_100_2	1.29	0.71	2.15	1	21.6	**0.000**	Low forest cover
	Fish presence	−2.66	−7.41	−0.20	1	4.6	**0.035**	Without fish
	Connectivity_100m	1.72	0.47	3.40	1	7.8	**0.007**	Many surrounding wetlands
	Local_1	−0.93	−2.18	0.03	1	3.6	0.063	Low aquatic pollution
	Local_2	1.95	0.70	3.96	1	11.2	**0.001**	Large, deep wetlands
	Local_3	1.26	0.35	2.54	1	8.1	**0.006**	Abundant aquatic vegetation
	Residuals				64			
b. *Lissotriton vulgaris*								
	Past_500_1	−0.78	−1.36	−0.28	1	9.8	**0.003**	Low urbanization in the past
	Past_500_2	−0.08	−0.51	0.35	1	0.1	0.710	
	Landscape_100_1	0.01	−0.39	0.42	1	0.0	0.980	
	Landscape_100_2	0.65	0.36	0.92	1	18.2	**0.000**	Low forest cover
	Fish presence	−1.11	−2.56	0.00	1	3.8	0.055	Without fish
	Connectivity_100m	0.69	0.12	1.30	1	5.6	**0.021**	Many surrounding wetlands
	Local_1	−0.81	−1.31	−0.36	1	13.0	**0.001**	Low aquatic pollution
	Local_2	0.85	0.41	1.32	1	15.2	**0.000**	Large, deep wetlands
	Local_3	0.67	0.28	1.10	1	11.8	**0.001**	Abundant aquatic vegetation
	Residuals				64			

(a) The crested newt Triturus cristatus and (b) the smooth newt Lissotriton vulgaris.

Bold values represent significant differences (α = 0.05). See Table 2 for details on the variables.

The best model for *L. vulgaris* (i.e. the model with rank 1 in Table 5) was very similar to the best model for *T. cristatus* (Table 3). *Lissotriton vulgaris* was related to present landscape features (scale, 100 m), past landscape (scale, 500 m), fish presence, local features and connectivity (scale, 100 m). Two further models showed weight greater than 0.1. All models with high support were similar, being subsets of the best model (Table 5). All models with weight greater than 0.1 included local features, connectivity and present and past landscape composition. Examination of variables included in the best AIC model showed that smooth newts were associated with large, deep and less polluted ponds with abundant aquatic vegetation, surrounded by a high number of wetlands within 100 m, in landscapes that are currently open and that had low urbanization in the past (Figure 3; Table 4b).

**Table 5:** Candidate models explaining spatial variation in abundance of *Lissotriton vulgaris* on the basis of ecological variables.

Rank	Model structure	K	Q-AICc	weight
1	Present landscape (100 m), past landscape (500 m), fish presence, suitability of pond features, connectivity within 100 m	10	68.21	0.393
2	Present landscape (100 m), past landscape (500 m), suitability of pond features, connectivity within 100 m	9	69.25	0.234
3	Present landscape (100 m), past landscape (500 m), fish presence, suitability of pond features, connectivity within 500 m	10	70.82	0.106
4	Present landscape (100 m), past landscape (500 m), suitability of pond features, connectivity within 500 m	9	70.97	0.099
5	Present landscape (100 m), past landscape (500 m), fish presence, suitability of pond features	9	71.03	0.096
6	Present landscape (100 m), past landscape (500 m), suitability of pond features	8	73.17	0.032
7	Present landscape (100 m), fish presence, suitability of pond features, connectivity within 100 m	8	73.29	0.031

Only models with weight >0.01 are shown here. K = number of estimated parameters.

The distribution of *T. cristatus* was nested within the distribution of *L. vulgaris*, as the smooth newt was detected in 11 out of the 12 ponds with crested newt. We therefore compared the features of ponds hosting both species with those of ponds with *L. vulgaris* only. Ponds with both species were significantly different for components 2 and 3 of the PCA run over pond features (*t*_26.663_ =  −3.161, *P*<0.01 and *t*_27.045_ =  −2.566, *P*<0.05, respectively), indicating that these ponds were larger and deeper, with more aquatic vegetation than those with *L. vulgaris* only (Table 6). The other environmental variables were not significantly different between the two groups of ponds (all *P*>0.11; Table 6).

**Table 6:** Comparison of local and landscape variables.

Species	Local_1	Local_2	Local_3	Wetlands100 m	Landscape100 m_2	Past Landscape500 m_1
*T.c*.+*L.v*.	−0.54±0.25	0.64±0.18	0.53±0.20	0.91±0.17	0.07±0.31	−0.30±0.21
*L.v.* only	0.01±0.18	−0.07±0.18	0.04±0.17	0.71±0.14	0.17±0.22	−0.06±0.23
*t*	1.673	−3.161	−2.566	−0.677	0.267	0.648
df	19.062	26.663	27.045	21.292	17.832	26.902
*P*	0.111	**0.004**	**0.016**	0.506	0.792	0.522

Data are shown for ponds with the smooth newt *Lissotriton vulgaris* (*L.v.*) only and those with both *L. vulgaris* and the crested newt *Triturus cristatus* (*T.c.*) (mean ± SE values unequal variance *t*-test). The “number of wetlands” were square-root transformed values; the other variables are components extracted by of PCAs: see methods for more details. Bold values represent significant differences (*α = *0.05).

## Discussion

### Commonness and Rarity

The analysis of ecological processes involved in the distribution of *T. cristatus* and *L. vulgaris* showed that, despite a difference in commonness, both species share similar responses to environmental features. As shown by the best AIC models (Table 4), all the processes tested had a similar influence on both species: their abundance was related to the same environmental variables, and the effect was in the same direction for both species. This indicates that conservation actions should consider multiple factors to adequately protect these species. The suitability of pond features (water quality, macrophytes, water depth), the absence of introduced fish species, the connectivity among sites, and the composition of present-day and historical landscapes were important for newt distribution. These processes are mostly determined by anthropogenic activities and are acting in a direction that is unfavourable for newt persistence, both in the study area and in most modern agricultural landscapes. It can therefore be expected that without action plans, both the rare and the “common” species will quickly decline. These results support recent evidence that not only the rarest, but also the apparently most common amphibians are at risk [10], [11], [17], [48]. Commonness patterns should not be overlooked in ecological research as commonness is only a transient state, which means that what is common today may be rare tomorrow [2].

The use of surrogate species has been a major tool in conservation planning, but has also received criticism as several conditions need to be met for applying conservation actions efficiently. Recent analyses showed empirical evidence for this concept, but more data are needed on amphibians [49]. Both newt species used similar habitats and responded similarly to disturbance, which supports the potential efficiency of the umbrella species concept in amphibians [3]. The similarity within several processes acting at multiple scales argues even more for simultaneous protection of common and rare species. Moreover, the rarest species responded more strongly to environmental changes than the most common species. Newt populations often exist within networks of meta-populations or patchy populations [50]–[52] and, in some cases, observed changes of occupancy may be part of extinction and colonization dynamics that are characteristics of meta-populations [53]; for instance, *L. vulgaris* was detected in one pond where it was not found 10 years before. Nevertheless, for *T. cristatus* the decline persists because of the overall loss of suitable habitats and, under situations of continuous loss of habitat quality, colonizations cannot compensate extinctions [17], [51]. Evaluating changes in occurrence across time gave coherent results with these patterns: in the recent surveys *T. cristatus* was not found again in half the ponds, whereas *L. vulgaris* was still found in all but two ponds. Such a focus has broad applications as both species are sympatric over a large part of their distribution ranges. However, targeting *T. cristatus* populations would favour only a small fraction of *L. vulgaris* populations, as they are six times more numerous than *T. cristatus* populations in the study area [22]. These results are confirmed with significant trends at a broader scale that showed that *T. cristatus* is more localized and less widespread than *L. vulgaris* [17]. In fact, actions on the ponds inhabited by the more common species could also help the recovery of *T. cristatus* in nearby populations as this species is favoured by a high density of ponds. On the other hand, our analysis considered species of newts typical of open landscapes, while responses to environmental modifications may be even more complex if the whole amphibian community is considered [22], [54], [55]. This means that the umbrella species approach should be used with care only once ecological requirements are sufficiently known.

### Ecological Processes: Shared and Specific Patterns across Studies

Previous studies on the ecology of crested and smooth newts considered heterogeneous sets of environmental variables and spatial scales [1], [15], [20]–[25], [39], [56] (Table 1). Each of these studies highlighted determinants of newt distribution and thus improved our knowledge in terms of conservation management. As outlined by Zanini et al. [57], there is geographic variation of underlying ecological processes and thus different results can be found in contrasted regions. For instance, Hartel et al. [21] indicate that traditional management of the landscapes studied in Romania was the basis of the relative unimportance of landscape determinants. The present study was conducted in an area where rarity was more pronounced than in other studies (but see [23]; Table 1). The results confirm previous knowledge on these species but also show that in altered landscapes, multiple processes are acting simultaneously on newt distribution. Indeed, all the processes considered contribute to explaining the distribution of the two species. In addition, considering different spatial scales and water pollution provided new insights into the anthropogenic pressure on natural populations.

### Towards Effective Conservation Measures

Although this study targeted areas that were known to be inhabited by a rare newt, both study species were absent from two-thirds of the ponds. The fact that this pastoral landscape hosts a high density of ponds [31] must therefore be balanced by their limited suitability for newt breeding. Although only one pond had disappeared since the last survey period (1990–2004), several ponds showed signs of future disappearance (i.e. shallow depth, eutrophication, partial destruction), suggesting a higher rate of pond loss over the long term, as shown in other studies [26], [58].

In terms of suitability of aquatic sites, ponds need to be restored to maintain a high water depth (i.e., at least 1 m in such agricultural lands) while avoiding fish introduction, as permanent ponds are more likely to sustain fish. Habitat restoration programs, including pond creations for *T. cristatus*, have proved to be successful and should be followed over wide areas [59]. Fish have been shown to be particularly detrimental to newts [20], [25], [60]–[62]. However, smooth and crested newts could also coexist with fish [1], [63], [64]. Here, introduced fish were found in 16% of ponds. Although most of them could not predate adult amphibians, they can eat eggs and larvae [65]. This could explain why we only found two populations of *T. cristatus* and four populations of *L. vulgaris* coexisting with fish. In these cases of cohabitation, newt abundance was very limited (maximum two *T. cristatus* and 17 *L. vulgaris* detected). Large fish were not found in coexistence with newts, except in one population of *L. vulgaris* where fish were not numerous. The highest susceptibility of crested newts to fish may be due to the more pelagic behaviour of its larvae [66], [67], but more work is needed to understand the mechanisms of coexistence between newts and fish [68]. Because amphibian resilience is possible after fish removal [69], this management action should be included in conservation plans. Aquatic vegetation provides support and protection for the eggs and shelter for the newts [15], [25], [70]–[72] and should be favoured, but without excess, as ecological succession would lead to pond disappearance [73]. Sources of pollution should be identified and managed to avoid run-off of pollutants. Too few studies have integrated pollution by fertilizers and domestic run-offs and how they affect amphibians [74], although laboratory experiments have shown their direct effects on larval stages [29]. The high concentration of pollutants found in ponds within this study and their relation to absence or low abundance of newts show that the landscape studied is heavily polluted. Buffer zones should therefore be designed around each pond to reduce the risk caused by the use of fertilizers and building water discharges.

Previous studies have shown that woods or scrubs should be maintained near breeding ponds as they provide an adequate space for vital activities such as feeding outside the reproductive season, but also for aestivation and wintering [28], [75], [76]. The preferential emigration from ponds is often toward forests instead of open landscapes in both *T. cristatus* and *L. vulgaris* [77], and the occupied ponds are usually only at a few hundred metres from forests [21], [22], [25]: these arguments further support the importance of forests. However, both *T. cristatus* and *L. vulgaris* typically breed in ponds located in open landscapes. The differences between the effect of past and previous landscapes showed that terrestrial processes are complex and would require specific investigations. Telemetry and capture-mark-recapture studies, such as those conducted by Jehle [28] are needed to understand terrestrial requirements in both traditional and modernized landscapes.

Connectivity, often highlighted in newt research [1], [20], is not only important at large scales around core ponds, but also in the vicinity given that the number of ponds within a radius of 100 m had a significant effect on both species in the present study. This confirms results of radio-telemetry showing that most individuals remain very close to the breeding ponds [28]. Viability prediction models highlighted that *T. cristatus* populations harbouring more than 40 adults may have a lower risk of extinction in case of isolation [78]. The usual number of adults detected in all the populations studied was below this value, emphasizing the importance of maintaining landscapes with high pond density.

In conclusion, commonness should not be neglected in conservation management and the adequacy of surrogate species should be evaluated to ensure that what is common now does not become rare. In the current perspective of amphibian decline, multiple stressors should be considered together to allow efficient conservation programs.
